# Crop prices, farm incomes, and food security during the COVID‐19 pandemic in India: Phone‐based producer survey evidence from Haryana State

**DOI:** 10.1111/agec.12633

**Published:** 2021-05-25

**Authors:** Francisco Ceballos, Samyuktha Kannan, Berber Kramer

**Affiliations:** ^1^ International Food Policy Research Institute (IFPRI) Markets, Trade, and Institutions Division Washington District of Columbia USA; ^2^ International Food Policy Research Institute South Asia Regional Office Delhi India

**Keywords:** agricultural income, COVID‐19, India, price risk, risk coping strategies

## Abstract

In March 2020, India declared a nationwide lockdown in response to the COVID‐19 pandemic. Such restrictions on mobility interrupted the normal functioning of agricultural value chains. For a sample of 1767 tomato and wheat producers in the state of Haryana, we study to what extent the lockdown limited access to inputs, labor, machinery, and markets to produce, harvest, and sell their crops. We quantify crop income reductions during the first months of the lockdown and analyze to what extent these are associated with borrowing and food insecurity. We find that wheat producers, for whom state‐led procurement guaranteed market access at fixed prices, suffered minimal declines in income. For tomato producers—an already more vulnerable population—income fell by 50% relative to their expected income in a normal year, largely due to a steep fall of tomato prices as they shifted from wholesale markets to local retail markets, resulting in a sharp increase in local supply. Relative to wheat producers affected by the lockdown, reduced income for tomato producers was associated with an increase in borrowing and reduced food security. We conclude that targeting producers of crops that face substantial price risk and introducing policies that stabilize market prices are important in efforts to aid recovery and build resilience of smallholder farmers.

## 1 INTRODUCTION

The COVID‐19 pandemic and the measures put in place to curtail it have taken a toll on economies worldwide. India, one of the largest economies in the world, implemented a strict lockdown starting March 2020. In April 2020, growth projections for the country were revised downward from 6% to 2%, with significant expected impacts on the agricultural sector from both demand and supply contractions (IMF, 2020). Such a downturn in economic activity would have major consequences for poverty reduction and food security across the country, as India's agricultural sector represents almost 15% of GDP and provides livelihoods for an estimated 126 million smallholder farmers (Bisht et al., 2020) and more than 100 million agricultural laborers and other value chain actors. As governments are being urged to provide relief to farmers and support their recovery (Narayanan and Saha, 2020), and as they are looking for ways to minimize the adverse consequences of policies to contain future outbreaks, it is imperative to understand how a lockdown affects agricultural value chains.

To provide a deeper understanding on the mechanisms at play and inform the design of recovery policies, we analyze how agricultural production and farmer livelihoods were disrupted during the lockdown. To do this, the paper aims to answer three main research questions. First, we aim to assess to what extent farmers’ crop income declined during the lockdown, focusing on two types of producers in the same geographical context: wheat producers, who were about to harvest their crops when the lockdown was announced and for whom policies were set in place to guarantee a market for their harvest; and tomato producers, who were amidst the growing season—and may thus have seen impacts to their production—and for whom no policies were set in place to support marketing. In this sense, we expect potentially larger adverse effects of the lockdown for tomato farmers. Second, in order to determine the extent to which farmers were able to absorb any resulting short‐term income shocks, we study whether reductions in agricultural income are associated with changes in borrowing and food insecurity, with potentially long‐term welfare effects. Third, we are interested in characterizing income reductions by farmer profiles; in particular, we analyze which farmer attributes help explain differences, if any, in income reductions across tomato and wheat producers.

We explore these questions by relying on survey data collected for 1767 wheat and tomato farmers spread across four districts in the state of Haryana, India. These surveys were conducted by phone during the lockdown in the context of an ongoing panel survey on agricultural risk management. In the case of wheat farmers, the phone surveys included a series of questions related to the effects of the lockdown on crop productivity, harvesting practices, harvest costs, and commercialization of produce after harvest. In the case of tomato, a multi‐picking crop, we administered the same set of questions through several follow‐up surveys over the course of the harvesting season, allowing us to explore the dynamic effects of the lockdown restrictions. In addition, for both wheat and tomato producers, we asked about borrowing and included a short module on household's access to food before and after the lockdown, providing insights on lockdown‐related borrowing and disruptions in food security. These data provide insights on the linkages between reduced agricultural income and risk coping behaviors.

We find important disruptions to agriculture during the lockdown, with income reductions varying across crops and over time. In the case of wheat, income reductions remain modest, as these farmers were able to reap the benefits of state procurement policies that allowed them to continue selling their harvest at minimum guaranteed prices. In the case of tomato, we observe higher income reductions across production, harvest, and post‐harvest stages. In addition, we observe major reductions related to the commercialization of tomato harvest; we estimate reductions of 50% relative to the expected income in a normal year, mainly due to output prices falling to about one third of farmers’ expected prices. Related literature and key informant interviews suggest that prices fell as traders, who normally serve to link farmers’ production with wholesale markets, could no longer travel or offered lower prices due to an increase in their operational costs (Varshney et al., 2020). As a result, farmers shifted to local retail markets, increasing local supply relative to demand and depressing prices for their produce. Reduced income is also associated with an increase in borrowing and reduced food security. This evidence highlights the severe consequences of a lockdown and associated market closures for the producers of this perishable horticultural crop and, in particular, the important role that price risk plays in securing profits from farming.

This paper contributes to the literature in three ways. First, our study relates to a growing literature analyzing the effects of the COVID‐19 pandemic and related restriction measures on agricultural livelihoods (Ceballos et al., 2020; Kumar et al., 2020; Rawal et al., 2020), agricultural value chains (Hailu, 2020), the agricultural grain (Brewin, 2020) and vegetable (Richards & Rickard, 2020) sectors, and food security (Abate et al., 2020; Alvi & Gupta, 2020). We add to this literature by quantifying reductions in income for producers of two different types of crops, wheat, and tomatoes, within the same region, and showing that the extent to which income was lowered varies widely across these two different value chains. To the best of our knowledge, none of the existing studies on the effects of the COVID‐19 pandemic and related restriction measures on agricultural livelihoods quantify income effects of the pandemic for different phases of production and marketing, while analyzing how the same lockdown in the same geographical context impacted producers of different types of crops.

Second, this paper relates to several strands of the agricultural economics literature around risk management and coping with shocks. Farmers generally have very limited options for avoiding pre‐harvest production losses (Moschini & Hennessy, 2001; Oerke & Dehne, 2004; Savary et al, 2012) or post‐harvest losses (Affognon et al., 2015; Hodges et al., 2011). In this light, rural household resilience to fluctuations in agricultural incomes is a key aspect for rural development. While the literature highlights various formal and informal coping mechanisms available to farmers (Dercon, 2002; Wik, 1999), these are generally limited and tend to be less available among the most vulnerable households (Gao & Mills, 2018; Harvey et al., 2014; Olsson et al., 2015). In this light, large reductions in income, like the ones we observe for tomato producers, can have major welfare implications in the long term; farmers may perceive an increase in future risk, potentially leading to ex ante under‐investment in profitable technologies (Cai et al., 2013; Cai et al., 2009; Cai, 2016; Cole et al., 2017; Karlan et al., 2014; Mobarak & Rosenzweig, 2012), and such a one‐time shock can have long‐term consequences on income paths and human capital development (Barrett & McPeak, 2006; Dercon & Hoddinott, 2004).

In this paper, we focus on a systemic shock. Studies that distinguish between idiosyncratic and systemic shocks find the former linked to household asset depletion as income smoothing strategy, and the latter as having more durable effects on consumption (Börner et al, 2015; Nguyen et al., 2020). This is to be expected since many of the informal coping strategies available to rural households (such as borrowing from local moneylenders or family and friends, or tapping into other sources of income like non‐agricultural labor) tend to fail when most households in an area are affected by the same shock (Dercon, 2002). Such insights are particularly important in the light of a global systemic shock like the COVID‐19 pandemic, which may bring about dire consequences in terms of food insecurity and nutrition. We find that in the short term, relative to wheat farmers, tomato farmers are more likely to borrow or become food insecure when suffering lockdown‐related reductions in agricultural income. Given this, it will be important to introduce recovery policies aimed at these farmers in order to avoid long‐lasting impacts on consumption.

Third, we contribute to a recent uptick in the literature around price risk in agriculture (Bellemare et al., 2020; Boyd, 2020; Boyd & Bellemare, 2019). Studies investigating farmers’ stated perceptions of risk find that market risk, including price risk, is one of the most significant risks to farmers (Duong et al., 2019). This is important in the light of an increase in food price volatility in the last decades, with recurring periods of price depression (FAO, 2018). Consistent with this literature, the main factor reducing agricultural income in the context of our study has been a fall in tomato prices. We conclude that policy efforts to provide disaster relief and build resilience should take into consideration the differential impacts of disruptions to agricultural markets on farmer livelihoods, with a need to focus on producers of crops exposed to price risk. Moreover, future policies for building resilience should focus not only on production risk management but, importantly, set price risk reduction as a key priority moving forward.

The paper proceeds as follows. Section 2 describes the study context and ex ante hypotheses we set out to test. Section 3 describes the data sources involved and the data collection methods, including sampling and procedures around the phone‐based interviews. Section 4 presents the results and Section 5 concludes, together with implications for policies around recovery.

## 2 CONTEXT AND HYPOTHESES

Late March 2020, as COVID‐19 had started spreading across India, a nationwide restriction to the movement of goods and people was instituted on account of mounting fears around a rapid spread of the pandemic and the country's vulnerable health system—with insufficient capacity to meet the projected demand for health care given its high population density. Restrictions were introduced just before the rabi (winter) season harvest window for many crops. Shortly after, the government relaxed some of these measures for a number of essential agricultural activities, including farming operations, input production, and commercialization, intra‐ and inter‐state movement of sowing and harvesting machinery, and procurement of agricultural commodities. During April and May, the government further extended these exemptions to other actors of the agricultural value chain.

Despite the measures to limit the effect of the lockdown on agricultural activities, numerous obstacles disrupted normal harvest operations. While the national government had allowed for the normal operation of licensed market yards (mandis), where most agricultural produce is sold, many state marketing boards, in charge of running mandis, kept them closed during the first weeks of April (Narayanan, 2020). The inter‐state flow of agricultural goods and equipment also suffered from mandatory border checks and general confusion around the exact details of the policies put in place. Availability of agricultural labor was largely affected too. In anticipation of the lockdown, large urban‐rural migration flows occurred, with people from cities returning to their rural hometowns. This, together with severe restrictions to agricultural laborers’ mobility, resulted in harvest operations becoming directly dependent on the local supply of labor and machinery.

**FIGURE 1:**
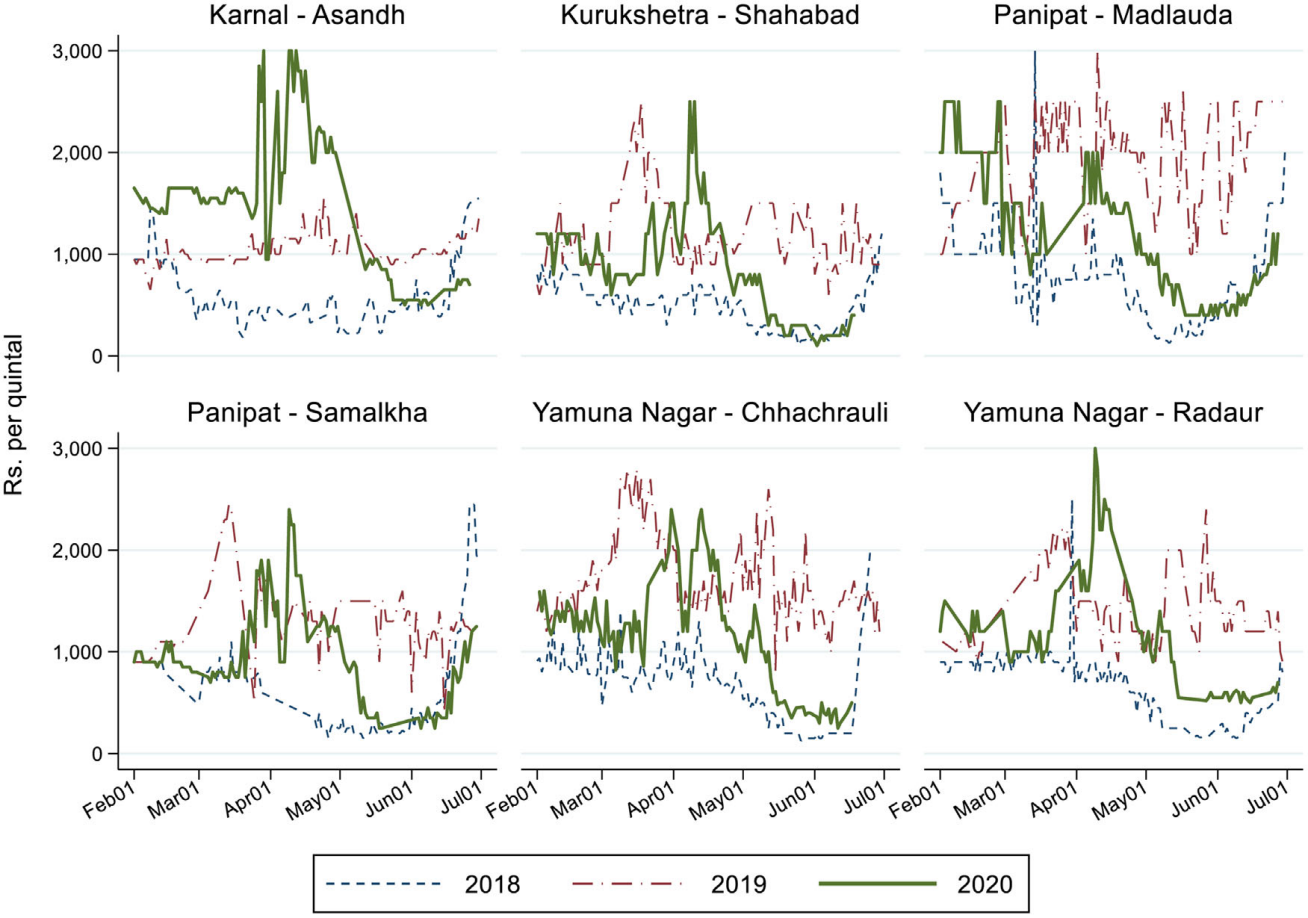
Tomato prices at selected markets in the study area [Color figure can be viewed at wileyonlinelibrary.com] *Note*: Daily prices at select official state markets in the study region. The data come from the Government of India's Agmarknet portal, https://agmarknet.gov.in/. Rs., Indian Rupees

In our study area, four districts in the state of Haryana, farmers were subject to significantly different policy contexts depending on the type of crop they cultivated. In the case of wheat, the state government imposed a staggered procurement system beginning on April 20th. Under such a scheme, wheat farmers could sell their harvest at the minimum support price (MSP) at licensed mandis, and the number of mandis was increased from 477 to 2000, with only 100 farmers allowed per day (Ceballos et al., 2020). The MSP at which wheat farmers could sell their harvest was Rs. 1925 per quintal, up from 1735 and 1840 in, respectively, 2018 and 2019. In contrast, in the case of tomato, no guaranteed public procurement scheme was available, and farmers had to sell their harvest at the running market price. Figure 1 shows the evolution of wholesale tomato prices around the rabi harvest season from 2018 through 2020 for markets in our four study districts. While prices initially increased (though becoming quite volatile) after the introduction of the lockdown measures in late March, these subsequently decreased until reaching similar levels to 2018 minimum prices by around mid‐May, when tomato harvest typically reaches its peak (Varshney et al., 2020). This price decrease seems to have been related to a lower presence of traders (*aarthiya*)—who normally act as middlemen to bring tomato harvest from the farmgate to regional markets—due to mobility restrictions or to them offering lower prices than normal. As a consequence, farmers shifted to local retail markets, increasing local supply and inducing downward pressure on prices.

This contrast between commercialization environments faced by wheat and tomato producers compounds with existing differences in farming practices and farmer characteristics (Table 1). While wheat is cultivated by a large majority of farmers in Haryana and generally considered to be a relatively safe crop, tomato is a high‐risk crop with higher costs of production and considerably more dependent on manual labor. In terms of farming activities, wheat is cultivated at a larger scale and typically harvested in one go using custom‐hired combine tractors, while smaller‐scale tomato fields undergo a series of pickings throughout the last 2 months of the growing season which are conducted exclusively by hand. Farmers cultivating these crops are also very different on average, with most tomato farmers not owning the land they cultivate (generally relying on sharecropping arrangements) and facing a far higher incidence of crop damage than wheat, particularly from pests and diseases. Since the national crop insurance scheme (Pradhan Mantri Fasal Bima Yojana, or PMFBY) does not cover tomato production and private insurance solutions are not widespread, tomato farmers also resort to very different coping strategies such as offering their labor or informal credit.

**TABLE 1:** Comparison of key tomato and wheat farming variables

	Wheat	Tomato
Cost of production	INR 11,949	INR 29,929
Median yield (quintals per acre)	20.0 quintals per acre	72.6 quintals per acre
Median expected price per quintal	INR 1925 per quintal	INR 1200 per quintal
Expected revenue per acre	INR 38,500 per acre	INR 87,120 per acre
Percentage farming on own land	82%	24%
Average area under crop	3.9 acres	1.6 acres
Percentage affected by crop damage in the last 5 years	22%	76%
Instance of crop damage due to pest and disease in the last 5 years	17%	67%
Average severity of damage due to pests and diseases	38%	54%
Finance operations through credit	Informal loans: 48% Formal loans: 21% Informal credit for inputs: 17%	Informal loans: 27% Formal loans: 3% Informal credit for inputs: 48%

*Note*: For most data reported in this table, we draw upon Ceballos, Kannan, and Kramer (2019). Median yields, expected prices, and thus expected revenue are based on the phone survey data collected during the lockdown and presented in this paper. For expected prices, wheat farmers report minimum support prices, whereas tomato farmers have likely reported prices in the best‐case scenario.

Based on these differences between crops, we hypothesize that the lockdown and related restrictions will have different effects for wheat and tomato farmers. First, given that tomato is a perishable crop, dependent on manual labor, and more exposed to damage from weather or pests and diseases, a reduction in the supply of labor, restricted access to inputs, and limited transportation around the lockdown should translate into higher pre‐ and post‐harvest crop losses. Second, while state government efforts to introduce a staggered procurement system with MSPs and an increased number of mandis may have benefitted wheat farmers, allowing them to secure the purchase of their harvest at the minimum support price, tomato farmers resorting to the open market may have had to accept similar or lower prices than previous years due to the lower presence of traders and the lower prices at local retail markets, which saw a sharp increase in supply. As a result, we expect larger decreases in agricultural incomes for tomato farmers than for wheat farmers, with an associated increase in borrowing, and, among those for whom borrowing may not be viable, a reduction in food security, with potentially harmful consequences for family members’ long‐term nutrition and health, in addition to future productivity.

## 3 DATA AND METHODS

**FIGURE 2:**
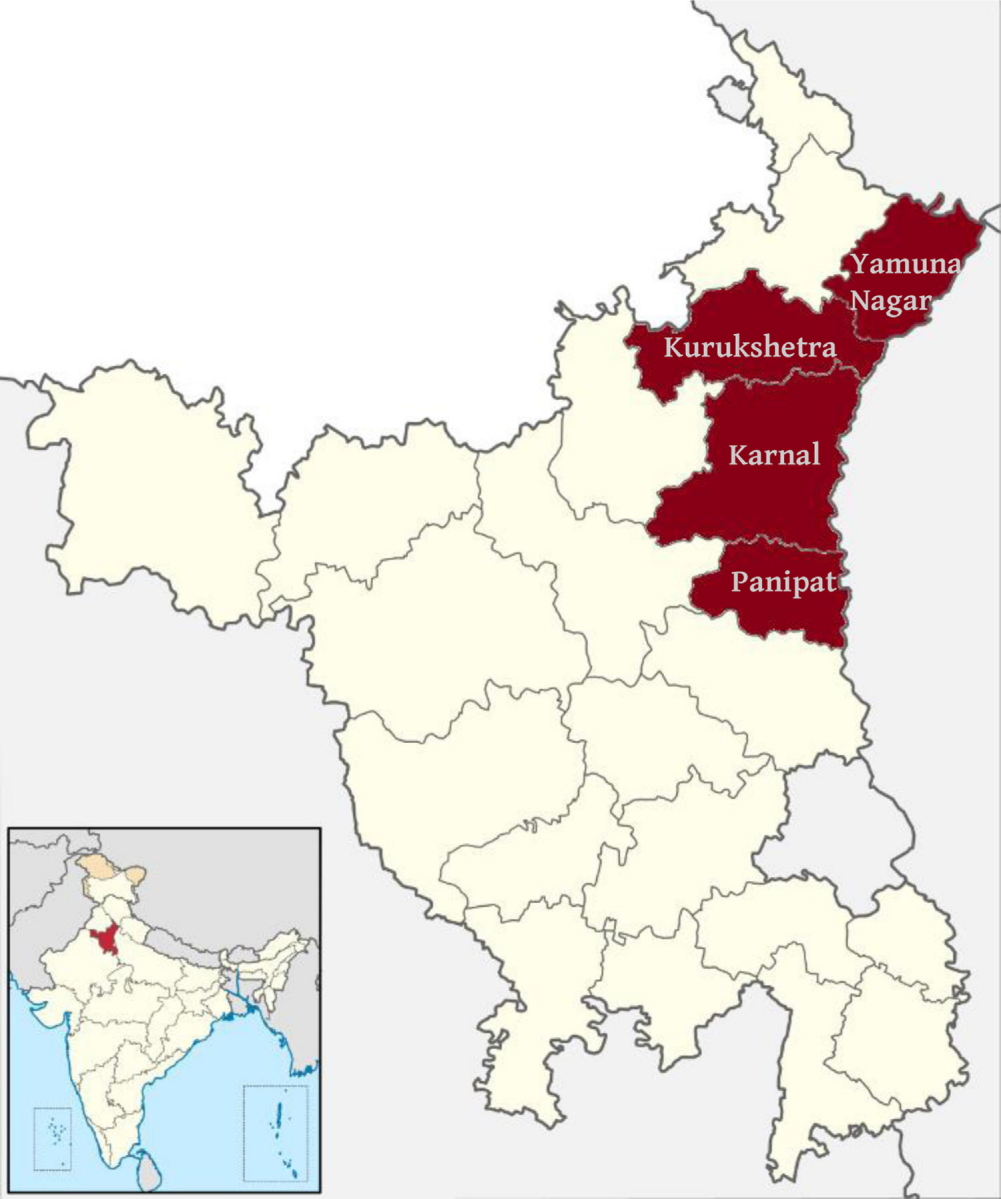
Map of the four study districts in Haryana state, India [Color figure can be viewed at wileyonlinelibrary.com]

This study relates to a broader ongoing impact evaluation to assess the effects of a novel risk management tool to protect farmers from agricultural production shocks: Picture‐Based Insurance (Ceballos, Kramer et al., 2019). As part of this study, the project is crowdsourcing images of farmers’ crops to monitor crop growth, management practices, and crop damage through a dedicated smartphone application named KisanCam. Participating farmers are spread across 101 villages in four contiguous districts: Karnal, Kurukshetra, Panipat, and Yamuna Nagar (see Figure 2). These villages belong to blocks having clusters of tomato producers and were identified by block‐level agricultural officers as those with higher concentrations of tomato growers.

The main data used in this paper come from phone interviews conducted from March to June 2020, after the COVID‐related lockdown measures went into effect. These surveys served as a follow‐up with participating study farmers close to the end of the rabi harvest season and was preceded by other in‐person data collection rounds in earlier seasons. The survey included a general module on farming activities, input use, and crop damage during the rabi season, followed by a COVID‐specific module on the effects of the lockdown on timing of harvest, marketing activities, and costs and availability of agricultural inputs, labor, machinery, and transport. In addition, a small module on food security before and after the lockdown was included, based on a modified and reduced version of the Household Food Insecurity Access Scale (HFIAS, Coates et al., 2007). Where available, we complement the data from these phone surveys with data acquired through the KisanCam application, and, as a robustness check, with data on caste collected as part of a baseline survey conducted between September and December 2018 for a small subsample of study farmers. Finally, we rely on key informant interviews with agricultural experts in Haryana to understand the overall scope and reach of the COVID‐related measures imposed in the state and to obtain context‐specific information regarding wheat and tomato harvesting and commercialization.

The remainder of this section provides more details on how we sampled farmers for the phone surveys, and how we construct our final analysis sample. Phone surveys were administered with two sets of farmers: all farmers who had participated in KisanCam during the rabi 2019–20 season, sending in images of their wheat and tomato crops (1722 farmers); and, as selection into the former sample could be influenced by variations in the program across villages,^1^ we also selected a random sample of producers from all 3367 farmers who had been invited to participate in KisanCam at the start of the rabi season. This sample of invited farmers was constructed to be as representative of producers in the study area as possible, while clustering on village and stratifying on quartiles for a farmer's operational land size (see Ceballos, Kramer et al., 2019).

Overall, we were able to interview 1275 wheat and 632 tomato farmers. The main reason for attrition was being unable to reach farmers (22% of wheat farmers and 7% of tomato farmers), followed by farmers not growing one of our targeted crops. Overall, however, these are high response rates for a phone survey, due to high levels of rapport between the survey team and study farmers, as well as a systematicc protocol to make multiple call‐backs at different times during the day.

Wheat farmers were interviewed only once, after harvest had been completed. Since tomato is a multi‐picking crop, each tomato farmer was asked a shorter questionnaire every one or 2 weeks over the course of the harvest window (April to June), with questions about timing, volume, costs, and commercialization (quantity and costs) of the produce in each picking. Once the farmer indicated that they had finished the last picking for the season, they completed the longer module that wheat producers had completed as well, inquiring about total production costs, crop damage, and coping mechanisms, and the COVID‐specific and food security modules. Of the 632 tomato farmers who completed the initial survey round, 492 farmers (78%) also completed the final survey round. Attrition in this case was associated with being a younger farmer, having a later tomato transplanting date, and reporting increased machinery costs in the first survey (Appendix Table 2). In what follows, we include only the 492 tomato farmers in our main analyses to ensure a consistent sample throughout the paper. To correct for attrition, we apply inverse probability weights to the sample (Wooldridge, 2002), in order to assign a higher weight to tomato farmers with initial characteristics similar to those who did not respond to the last survey.^2^ Table 2 summarizes the construction of our final analysis sample.

**TABLE 2:** Construction of our analysis sample

Data source	Wheat	Tomato
Farmers invited to participate in KisanCam	3367	
Farmers who participated in KisanCam (all contacted for phone survey)	1016	706
Total number of farmers sampled for phone survey	1865	706
Participated in at least one round of phone survey	1275	639
Complete data from last survey round/final analysis sample	1275	492

## 4 RESULTS

This section presents our results. We first provide summary statistics on the extent to which wheat and tomato farmers’ agricultural income was reduced because of the lockdown. Next, we analyze to what extent reductions in agricultural income are associated with borrowing and food insecurity. Finally, we analyze to what extent reduced agricultural income is predicted by a range of potential explanatory variables, including age, education, caste, landholdings, and harvesting dates, and whether differences between wheat and tomato farmers, if any, can be ascribed to these explanatory variables.

### 4.1 Agricultural income reductions for wheat and tomato farmers during the lockdown

Table 3 describes the ways in which the lockdown disrupted production, harvest, and marketing. For different types of disruptions from the lockdown, column (1) provides the proportion of wheat producers that reported being affected, and the reductions in crop income associated to each type of disruption (averaged across all wheat farmers). Similarly, column (2) summarizes the proportion of tomato farmers that reported being affected in at least one of the phone surveys, along with total associated income reductions as reported in the last survey round (except for increased transport costs and damage to tomatoes waiting to be sold, which were reported in each round, and thus we aggregate income reductions reported across phone survey rounds). Column (3) presents the difference between wheat and tomato farmers.

**TABLE 3:** Disruptions to agriculture and associated income reductions during the lockdown

	Wheat producers	Tomato producers	Difference	
	(1)	(2)	(3)	
**A. Production and harvest phase**				
Harvested earlier than planned (1/0)	0.109	0.023	–0.086	***
Harvested later than planned (1/0)	0.320	0.188	–0.132	***
Had difficulty accessing inputs (1/0)	n/a	0.463	n/a	
Spent more on labor (1/0)	0.230	0.312	0.082	***
Increased labor costs per acre (INR)	165.3	1673	1508	***
Spent more on machinery/equipment (1/0)	0.245	0.141	–0.104	***
Increased machinery costs per acre (INR)	140.0	689.2	549.2	***
Disruption in production/harvest phase (1/0)	0.558	0.639	0.081	**
Crop income reductions per acre (INR)	304.1	2362	2058	***
**B. Post‐harvest and marketing phase**				
Spent more on transport to the market (1/0)	0.153	0.087	–0.066	***
Increased spending on transport per acre (INR)	79.32	314.8	235.5	***
Stored harvest because had no market (1/0)	0.349	0.136	–0.213	***
Discarded/lost this harvest in storage (1/0)	0.013	0.069	0.056	***
Value discarded/lost in storage per acre (INR)	102.8	274.8	172.0	**
Sold harvest for less than expected (1/0)	0.002	0.468	0.466	***
Expected minus actual price per quintal (INR)	n/a	786.2	786.2	***
Expected minus actual revenue per acre (INR)	n/a	40,350	40,350	***
Disruption in post‐harvest phase (1/0)	0.165	0.518	0.353	***
Crop income reductions per acre (INR)	182.3	40,901	40,719	***
**C. Aggregated statistics**				
Reports any disruption (1/0)	0.605	0.754	0.149	***
Total crop income reductions per acre (INR)	486.5	43,241	42,755	***
Number of observations	1275	492	1767	

*Note*: This table includes two types of variables: dummy variables, which take on a value of one if the respondent reports the listed disruption, and zero otherwise (marked as "1/0"); and continuous variables, which are reported in Indian Rupees ("INR"). Continuous variables include all observations, including farmers who did not experience disruptions, for whom these variables take on a value of zero. For expected prices, we use the median expected price reported by farmers, and for actual prices, we use the average across pickings. Means for tomato farmers have been corrected for attrition using inverse probability weights.

* *p *< .05.

** *p *< .01.

*** *p *< .001, based on a *t*‐test for differences in means for continuous variables, and a χ^2^‐test for binary variables.

The lockdown started after the wheat growing season, right around harvest, and hence could not have had any effects on wheat production or input use. Nevertheless, given uncertainties around the commercialization environment and official procurement policy during the weeks around the lockdown, in addition to the staggered procurement scheme introduced to limit the agglomeration of people at mandis, a substantial number of farmers reported adjusting the timing of wheat harvest; 11% of wheat farmers harvested earlier than planned, and 32% harvested later than planned.^3^ We observe increased spending on harvest activities; 23% and 25% of farmers, respectively, reported spending more on labor and machinery than usual, with the average farmer spending an extra INR 165 in labor and INR 140 in machinery per acre cultivated. In other words, the lockdown represented only a small increase in harvest costs of INR 304 per acre for wheat farmers.

Given the timing of the lockdown, we would expect the greatest reductions in income for wheat farmers in the post‐harvest phase. Along these lines, fifteen percent of farmers reported spending more on transport to the market, but the average increase of INR 79 across wheat producers remains modest. Since wheat is not a perishable crop, even though about one third of farmers had to store harvest because there was no market, very few (1.3%) reported discarding or losing harvest in storage, with a total reported income reduction of INR 103. Finally, a negligible number of wheat producers reported selling their crop at a lower price because of the lockdown; farmers were able to take advantage of the state procurement system for wheat and received the minimum support price, eliminating potential price fluctuations. As a result, the total amount by which wheat income was reduced—at the time of harvest and in the post‐harvest phase—remains relatively limited at an estimated INR 487 per acre, or approximately 4% of cultivation costs.

For tomato farmers, results are strikingly different. With tomato harvest starting only one month after the lockdown, tomato farmers were less likely to adjust their harvest practices, with a negligible fraction harvesting earlier and 19% (or 13 percentage points lower than wheat farmers) delaying their harvest. Nonetheless, income reductions appear more severe for tomato farmers. Since the lockdown was announced during the tomato growing season, crop production could be affected, and 46% indeed reported having faced difficulties accessing inputs; particularly pesticides (100%) and fertilizers (46%). We did not directly capture damage from a lack of access to inputs, but find, in further analyses, that although farmers with damage had lower yields, differences were not statistically significant: among farmers who faced challenges accessing inputs, 97% reported damage to their crop, and those farmers harvested 77.4 quintals/acre, compared to 85.8 quintals/acre among farmers who did not report damage (*p* = .227). Tomato producers were also more likely to report an increase in labor costs, spending on average nearly INR 1508 more on labor per acre than wheat producers. In terms of spending on machinery, they were less likely to report an increase but reported an overall larger amount than wheat producers, with the lockdown increasing per acre costs of equipment for the average tomato farmer by an additional INR 550.

**FIGURE 3:**
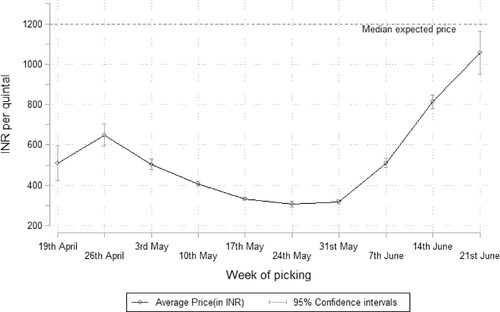
Average farmgate prices reported by tomato producers during the lockdown *Note*: Average farmgate prices as reported by surveyed tomato farmers by week of picking. The median expected price is calculated among farmers who reported selling their tomatoes for less than expected; the expected price is drawn as a reference point

In the post‐harvest and marketing phase, tomato farmers were less likely to face increased transportation costs than wheat farmers, but on average, their transportation costs increased more; not only because tomato farmers harvest in multiple pickings, and need to transport their produce to markets several times across the season, but also, as suggested by local key informants, because farmers were moving from itinerant traders at the farmgate to local markets in search of better prices. Overall, transportation costs increased by INR 235 more for tomato farmers than for wheat farmers, though this amount shows a large degree of variation between farmers. Since tomato is a perishable crop, farmers were less likely to store their harvest, but when doing so they were more likely to lose their harvest in storage than wheat farmers, with an estimated value of the associated income reduction of an additional INR 172 relative to wheat farmers. Although sizeable, these figures are dwarfed by the extent to which tomato producers’ income reduced due to a fall in prices. Nearly half sold their harvest at lower prices than what they expected to receive, and on average, actual prices were far below the median farmers’ expected prices of INR 1200 per quintal (see Figure 3). As a result, average tomato production income was reduced by a whopping INR 40,350 per acre.

All in all, the lockdown—and especially the associated fall in prices—had severe economic consequences for tomato farmers. When combining income reductions from disruptions during production and harvest (Table 3 Panel A) with the post‐harvest related disruptions, but not yet considering reduced income due to lower than expected prices (first two categories in Table 3 Panel B), the average farmer already reported total income reductions of INR 2891 per acre. This is nearly six times the average amount by which wheat income reduced, and close to 10% of their normal cultivating costs; even though not yet considering the reduced income from the steep drop in prices. When considering the prices at which they were able to market their harvest, farmers’ income reduced on average by INR 43,241 per acre, or nearly 1.5 times the production costs and half of the farmer's expected revenue of INR 87,120 per acre in a normal year. Income reductions of this magnitude will have largely eliminated profit margins, and although the average wheat farmer cultivates more acres than the average tomato farmer, total income will have reduced more not only per acre but also per farmer among tomato farmers, given the stark differences in reduced income per acre.

**FIGURE 4:**
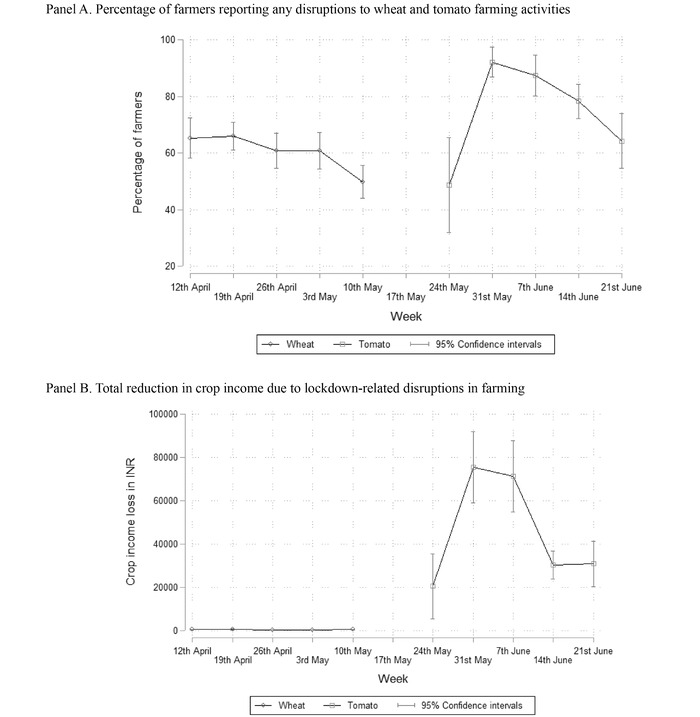
Disruptions related to lockdown for wheat and tomato producers *Note*: Proportion of farmers that reported at least one disruption in production and related activities (Panel A), and estimated reduction in crop income (Panel B). For tomato farmers, we aggregate reductions in income reported throughout the season and report these under the week during which the farmer completed his final interview

Figure 4 summarizes our main outcome variables by survey week. Panel A reports the proportion of farmers that reported disruptions to production or harvest in a given week. Panel B provides average reductions in crop income associated with these disruptions. For tomato producers, we assign a farmer to the week in which that farmer completed the last phone survey. While disruptions for wheat farmers do not seem to change much over time, tomato farmers finishing their harvest relatively early in the season (i.e., before the end of May) or relatively later in the season (i.e., towards the end of June) were significantly less likely to report disruptions, and this is associated with significantly lower estimated reductions in tomato income. In particular, income reductions were greatest for farmers who completed harvest towards the end of May or early June, as they harvested the majority of their tomatoes during the period that prices were at their lowest levels, yielding at most INR 400 per quintal or only one third of the median expected price.

Such a sharp fall in prices seems to have been directly related to the impacts of the lockdown. Normally, tomato producers sell their tomatoes at the farmgate to itinerant traders (*aarthiya*), who in turn commercialize the produce at major urban wholesale markets in the state or across state borders. The travel and transportation restrictions impeded the presence of *aarthiya* coming from other districts and states, made it more difficult for *aarthiya* to take produce across state borders, and increased transportation costs, reducing *aarthiya*’s willingness to pay for the produce. Moreover, a fall in the institutional demand for tomatoes due to the disruption of normal business activities (Pingali & Mittra, 2020) may have further lowered prices for farmers serving wholesale markets. As a result, farmers would have had to either accept the lower prices offered by *aarthiya* or shift to selling in local markets, where indeed supply of tomatoes increased relative to markets located at larger urban centers (Varshney et al., 2020). Under a relatively stable demand in these local markets, this would have put downward pressure on prices.

### 4.2 Association between agricultural income reductions, food insecurity, and borrowing

In this section, we analyze to what extent reported reductions in crop income during the lockdown are associated with borrowing and food insecurity, as an indication of whether farmers whose incomes reduced had to resort to costly coping strategies or were forced to reduce food consumption as a result of the income shock. While other formal or informal risk‐coping strategies such as resorting to savings or selling assets are possible, questions around these were not included to limit the length of the phone survey. We decided to focus on borrowing since it can bring about over‐indebtedness, with long‐term welfare implications, such as an increase in poverty or reduced future risk‐coping capacity (Chichaibelu & Waibel, 2018; Mutsonziwa & Fanta, 2019; Schicks, 2013). Moreover, at baseline, borrowing was found to be the main risk‐coping strategy reported by study farmers, and over‐indebtedness could risk farmers’ ability to invest in future agricultural seasons, since credit is also one of the main ways through which farmers finance their farming activities (Ceballos, Kannan et al., 2019).

Table 4 first indicates the proportion of respondents that needed to borrow to cope with the income reductions reported in Table 3, followed by the proportion of farmers that did not face food insecurity before the lockdown but did become food insecure at least once during the lockdown. In the case of the latter, we asked farmers about food insecurity experiences at any point during the month (i) before the start of the lockdown and (ii) before the interview (that is, during the lockdown); we respectively refer to these as the months "before the lockdown" and "during the lockdown" for clarity.^4^ In Appendix Table 1, we report the proportion of respondents that reported facing each of the three types of food insecurity, separately for the periods before and during the lockdown.^5^

**TABLE 4:** Borrowing and food security during the lockdown

	Wheat producers	Tomato producers	Difference	
	(1)	(2)	(3)	
Had to borrow to finance agricultural income losses from lockdown	0.016	0.083	0.067	***
Number of observations	1274	492		
Food insecure during, not before, lockdown				
Cannot afford sufficient quantity	0.005	0.000	–0.005	*
Cannot afford sufficient variety	0.015	0.004	–0.011	*
Cannot access sufficient variety	0.214	0.193	–0.021	
Any food insecurity experience	0.223	0.195	–0.028	
Number of observations	1162	483		

*Note*: Proportion of farmers that reported having to borrow to cope with crop income losses due to lockdown and that reported experiencing a given food insecurity experience "rarely", "often", or "frequently" at any point during the month before the interview (during the lockdown), but not during the month before the lockdown. Means for tomato farmers have been corrected for attrition using inverse probability weights. Column (3) indicates statistical significance from unpaired *t*‐tests for differences between wheat and tomato farmers,.

* *p *< .05.

** *p *< .01.

*** *p *< .001.

We observe significant differences between wheat and tomato farmers. Whereas only 1.6% of wheat farmers had to take out a loan, a significantly larger 8.3% of tomato farmers resorted to borrowing (*p *< .001), consistent with our finding that the latter suffered greater reductions in income. Focusing on changes in food security, we do not find stark differences between wheat and tomato farmers. We find negligible changes in the proportion of wheat and tomato farmers who could no longer afford a sufficient quantity or variety of food at conventional significance levels, although it is important to stress that these are short‐term changes and that agricultural income reductions may affect budgets and hence affordability in the longer term. The lockdown did nonetheless bring about significant disruptions in terms of farmers’ ability to access a sufficient *variety* of foods among both wheat and tomato farmers; 21.4% of wheat farmers became food insecure in this regard, and in the case of tomato farmers, this was 19.3%. Overall, when focusing on the aggregated variable capturing whether a household experienced at least one of the three types of food insecurity after (but not before) the lockdown, 22.3% of wheat farmers became food insecure at some point, which is not significantly different from the 19.5 figure for tomato farmers.

In Table 5, we explore whether borrowing and food insecurity after the lockdown is associated with reductions in crop income. We regress indicators for whether the farmer had to borrow (columns 1–2) and for whether the farmer became food insecure after the lockdown (columns 3–4) on a variable that indicates tomato producers ("Grows tomato"), variables related to crop income reductions during the lockdown, and interaction terms with the first variable ("…X Grows tomato"), controlling for block fixed effects to capture heterogeneity across regions. Even columns use binary variables for whether a farmer's income reduced, whereas odd columns use an ordinal variable with six values to indicate the size of the reduction.^6^ The aim is to test whether those producers with (greater) reductions were more likely to borrow or become food insecure, but given that the model does not include a complete set of covariates that can influence the decision to borrow or reduce consumption, these estimates cannot be interpreted as causal effects of the lockdown.

**TABLE 5:** Association between reduced income, borrowing, and food insecurity

Dependent variable	Had to borrow		Food insecure after lockdown	
Type of variable for reduced crop income	Binary (any income reduction) (1)	Ordinal (amount by which reduced) (2)	Binary (any income reduction) (3)	Ordinal (amount by which reduced) (4)
Grows tomato	0.004	–0.008	–0.043*	–0.095***
	(0.013)	(0.014)	(0.018)	(0.026)
All disruptions	–0.001	0.002	–0.114***	–0.075***
	(0.012)	(0.005)	(0.030)	(0.013)
… X Grows tomato	0.087**	0.023**	0.101*	0.084***
	(0.027)	(0.008)	(0.039)	(0.016)
Constant	–0.011	–0.010	0.326***	0.335***
	(0.010)	(0.008)	(0.045)	(0.042)
R‐squared	0.073	0.080	0.321	0.337
Number of observations	1750	1750	1634	1634
Number of clusters	100	100	100	100

*Note*: Coefficients estimated using an ordinal least squares model controlling for block fixed effects (not reported), with standard errors clustered at the village level. Coefficients for tomato farmers have been corrected for attrition using inverse probability weights. Food security observations are missing for 110 wheat producers and nine tomato producers due to a change in the survey instrument.

* *p *< .05.

** *p *< .01.

*** *p *< .001.

Column 1 shows that the average tomato farmer is more likely to borrow than a wheat farmer, but only if having experienced disruptions. Likewise, column 2 shows that the probability of having to borrow is increasing in the magnitude by which crop income was reduced, but only among tomato farmers. On average, wheat farmers experiencing disruptions to their crop income are less likely to become food insecure, but we do not observe this consistently among tomato farmers (columns 3–4); for them, relative to wheat farmers, disruptions are associated with an increase in food insecurity. In additional analyses (not shown for brevity), we find that this is the case especially during the post‐harvest and marketing phase, where tomato farmers experienced the greatest reductions.

Summarizing, relative to wheat farmers, we find that among tomato farmers, crop income reductions are associated with a significant increase in the probability of borrowing and becoming food insecure.

### 4.3 Determinants of agricultural income reductions for wheat and tomato farmers

In a final analysis, we focus on the question whether reduced crop income is associated with different types of farmer characteristics, and if so, whether differences between wheat and tomato farmer characteristics can explain the observed differences in income reductions between the two. To that end, Table 6 presents estimates from a linear regression model for lockdown‐related reductions in crop income as a function of a binary variable that indicates farmers who were growing tomatoes during the rabi season, while controlling for block fixed effects (to capture potential heterogeneity in the impacts of the lockdown at the administrative level) and other potential explanatory variables.^7^ We focus on income reductions aggregated across the season in columns 1–2, on those experienced during production and harvesting in columns 3–4, and on reduced income due to factors in the post‐harvest and marketing phases—including the consequences of the fall in tomato prices—in columns 5–6. Standard errors are clustered by block.

**TABLE 6:** Association between income reductions, borrowing, and food insecurity

	Crop income reductions: Season total		Crop income reductions: Production, harvest		Crop income reductions: Post‐harvest, marketing	
	(1)	(2)	(3)	(4)	(5)	(6)
Grows tomato	43,826***	42,843***	2045***	2022***	41,781***	40,820***
	(5531)	(5517)	(377.5)	(388.1)	(5612)	(5601)
Farmer age—lowest tercile (18–35 years)		658.8		83.43		575.3
		(2225)		(151.1)		(2284)
Farmer age—highest tercile (49–83 years)		1605		–123.1		1,728
		(2292)		(153.7)		(2337)
Medium education level		2718		–183.1		2902
		(2251)		(124.4)		(2287)
High education level		–212.2		–416.4*		204.2
		(2720)		(166.7)		(2771)
Above‐median landholdings		–66.42		–57.13		–9286
		(1391)		(93.30)		(1386)
Harvested after median harvest date		–4284		–386.1		–3898
		(3415)		(196.7)		(3461)
Caste		–2770		473.8		–3423
		4976		(272.5)		(4980)
Number of observations	1750	1750	1750	1750	1750	1750
R‐squared	0.310	0.316	0.215	0.210	0.286	0.290

*Note*: Coefficients estimated using an ordinal least squares model controlling for block fixed effects (not reported), with standard errors clustered at the village level. For variables with missing values, we impute missing values with the variable average (continuous variables) or zeros (dummy variables) and include for each of these variables a dummy that takes on value one if a value was imputed (and zero otherwise). Coefficients for tomato farmers have been corrected for attrition using inverse probability weights.

* *p *< .05.

** *p *< .01.

*** *p *< .001.

In odd‐numbered columns, we only control for block fixed effects and do not include any other variables. In line with the difference observed in Table 3, tomato producers lose a significant INR 43,826 more due to the lockdown compared to wheat producers (*p *< .001), indicating that the differences observed are not explained by an imbalance of wheat and tomato producers across blocks. We do, however, observe significant variation in the magnitude of income reductions across blocks, even within the same district. The greatest reductions are observed in Shahbad and Sadaura blocks, from respectively Kurukshetra and Yamuna Nagar districts, and the smallest reductions are observed in Ladwa and Nilokheri blocks, from Kurukshetra and Karnal districts. Combined, this regression model with block fixed effects and an indicator for farmers growing tomatoes is able to explain more than 30% of the variation in reductions in crop income.

In even‐numbered columns, we add a number of demographic and agricultural covariates, including a farmer's age category (distinguishing younger and older farmers from our reference group of middle‐aged farmers), level of education (using farmers with lower levels of education as the reference group), a variable indicating above‐median operational land holdings, a variable indicating whether the farmer harvested after the median harvest date in the sample, and a variable indicating whether a farmer belongs to a lower caste, which is more common among tomato farmers, and comes with relatively less social capital, for instance in terms of access to social networks and informal safety nets (Ceballos, Kannan et al., 2019).^8^ The survey did not focus on collecting an extensive set of control variables that may have provided for a more complete model, such as distance to the nearest wholesale and retail market, marketing channels and farmer wealth, limiting the scope for causal inference.

We do not observe significant differences in total reductions in crop income per acre depending on a farmer's age, level of education, landholdings, harvesting dates, or caste. Income reductions from factors in the production phase are decreasing in a farmer's level of education and age, with income for the highest‐educated farmers reducing by INR 416 less than for the least‐educated farmers (*p *< .05). By contrast, income reductions due to post‐harvest and marketing factors are increasing in farmer's age and level of education, although these are not statistically significant. In general, harvesting dates do not seem to determine the level of income reductions. Importantly, including controls does not reduce the size or significance of the coefficient on the variable "Grows tomato". This means that the estimated difference in reductions in crop income between wheat and tomato producers cannot be accounted for by any of the variables that we include as covariates in our analyses. Although we do not intend to make any causal inference, this persistence of the gap between tomato and wheat producers suggests that farmers may have suffered more solely because they were growing tomatoes, with its associated attributes and context; and not because they differ from wheat farmers in observable ways.

## 5 CONCLUSION

Global crises can have major consequences for rural economies. This paper analyzes disruptions to wheat and tomato farming activities during India's national lockdown around the global outbreak of COVID‐19. In March 2020, India instituted widespread restrictions to the movement of goods and people, affecting economic activities at an unprecedented scale, just as farmers had started preparing for the harvest season and would soon need to start marketing their produce. We present novel primary evidence on the extent to which crop income reduced as a result of disruptions to agricultural production during the lockdown. We distinguish between income reductions from factors that arose in the production and harvest phases versus income reductions from issues experienced post‐harvest and at the time of marketing. We also test whether these reductions in crop income are associated with borrowing and food insecurity, and we explore whether variation in reduced crop income within our sample is absorbed by variation in a select set of geographical, demographic, and agricultural characteristics that were available from the surveys.

Our analyses rely on detailed phone survey data for 1767 farmers across four districts in the state of Haryana, India. The phone surveys were designed to collect data that would allow us to analyze disruptions to agricultural activities across two separate value chains—wheat and tomatoes—with differences in institutional arrangements between the two. Wheat farming is highly mechanized, faces relatively low production risk, and relies on an established procurement system run by the state government which guarantees a minimum support price for the harvested crop. Moreover, the lockdown was announced as the wheat growing season had come to an end and farmers were preparing for harvest, minimizing impacts on productivity. Tomato farming, on the other hand, is generally done by relatively less wealthy farmers with smaller landholdings and less access to formal risk management instruments; tomato farming depends considerably more on manual labor, which became scarce during the pandemic; and it is exposed to higher production risks, with the lockdown starting amidst the tomato cultivation period. On top of that, tomato harvest is generally sold in the open market at prices that fluctuate wildly even within a given season.

We find minor disruptions reported by wheat farmers, in the form of a slight increase in labor and machinery costs around harvest, and transportation of their grains to the market. Overall, the additional costs brought about by the lockdown amount to about 4% of the cultivation costs for wheat on a per acre basis. One explanation for why income did not reduce much among wheat farmers is that the state instituted a policy that allowed farmers to continue selling their wheat at the prevailing guaranteed prices. The story is however very different for tomato farmers. Income reductions from increased production costs, harvest costs, and transportation to the market alone are nearly six times higher than those for wheat, and nearly 10% of total cultivation costs per acre. The main disruption, however, comes in the form of the very low prices at which tomato farmers could sell their produce. We estimate that the average tomato farmer in our sample was able to obtain only one third of their expected revenue from one acre. All in all, total estimated income reductions per acre for the average tomato farmer amount to more than INR 43,000, or about 50% of the expected income in a normal year.

Although we do not intend to make any causal inference, we further show that these reductions in crop income can have important consequences for farmers in our sample. Relative to wheat farmers, reduced tomato income is associated with higher borrowing rates and higher levels of food insecurity among those affected. Prior literature has shown that such strategies can have long‐term effects on the level of indebtedness and future income flows, with potential consequences on future food security and human capital accumulation (Barrett & McPeak, 2006; Dercon & Hoddinott, 2004). In addition, we show that the difference in reductions in wheat versus tomato income is not driven by differences in location or farmer characteristics such as age, education, landholdings, or caste. This evidence seems to support the claim that tomato farmers suffered more than wheat farmers mostly because there was no policy in place to support marketing of this perishable crop. In this sense, their continued reliance on fluctuating open market prices will make these farmers extremely vulnerable to future systemic shocks and crises, including future lockdowns to contain further outbreaks of COVID‐19 or other infectious diseases.

Our study subjects are participating in a broader project that is testing a novel insurance product, covering farmers for production damage that is visible in pictures of insured crops, sent in on a regular basis over the growing season. While products such as these can protect farmers against physical crop damage, little to no instruments are available to shield farmers from post‐harvest shocks and, most importantly, the risk of price fluctuations. We provide evidence of the direct income reductions from price fluctuations being much larger than those stemming from other hazards, reducing income by nearly 50% of the expected income in a normal year. This is likely an underestimation of the total economic cost for those involved in tomato farming, given that an increase in perceived risk will be associated with lower investments in such profitable yet high‐risk agricultural activities in the future (Elbers et al., 2007).

Overall, our findings point to the fragility of procurement mechanisms available to farmers growing perishable commodities, outside of the main cereals, pulses, and oilseeds covered under government procurement schemes. While *aarthiya* fulfill relevant roles in these commodities’ value chains—by providing farmers with a secure channel to sell their produce, linking them with larger markets with sufficient demand, and providing them with working capital—, some critics argue against their excessive power and the potential for corruption, since the proceeds from the sale of produce are routed back to the farmers through them.^9^ The evidence presented in our study, however, points to their importance in maintaining farmers’ incomes, at least in the *status quo*. In this sense, further research is warranted to provide a deeper understanding of the advantages and disadvantages of the current *aarthiya* system, as well as to explore any viable alternatives, in building farmers’ resilience.

Another relevant research avenue is to devise functional schemes to help farmers cope with price risk. Efficient price support schemes are rare, even in developed countries.^10^ India's minimum support price scheme is limited to a small basket of crops, can only handle limited volumes of production (beyond which the price is not guaranteed), induces distortions in agricultural production, and is costly to administer (Gulati et al., 2018). Expanding the scheme to commodities like tomato could reduce inequality between producers of different types of crops, and has been proposed in the past,^11^ but concerns remain around standardization stemming from the large variability in size, quality, and diverse varieties that arrive to markets. India has also attempted expanding price guarantees through the national government Market Intervention Scheme (MIS) and Haryana's state government Bhavantar Bharpayee Yojana (BBY), but these schemes have limited reach and benefits in practice.^12^

Alternatively, public‐private partnerships may offer a solution. Contract farming agreements that provide farmers with a fixed price for their harvest could be viable, though they are hard to coordinate when the farmer base is atomized and sporadic and may exclude *force majeur* events such as a pandemic. Fostering supply chain modernization through, for instance, direct supermarket procurement could also help improve prices and reduce price risk (Nuthalapati et al., 2020). In addition, private index insurance products that provide a payout when the local market price (for a reference variety and quality) falls under a minimum threshold, without provisions for procuring the product itself, may be a potential solution to the problems associated with price guarantee schemes, by providing additional income to farmers during generalized price downfalls, yet avoiding any direct market intervention. While a few similar products have been tried in small pilot implementations (Karlan et al., 2011; Shee & Turvey, 2012), affordably covering the catastrophic risk layer of such a product in a commercial way (potentially involving private commodity markets) remains a challenge and entails an open research question.

## Supporting information

Appendix Table 1—Disaggregated food insecurity experiences before and during the lockdown
Appendix Table 2—Determinants of attrition in tomato farmers (probit)

## Footnotes

1 The type of incentive for sending in images has been randomized at the village level: in 50% of the sample, farmers sending in images are rewarded with a cash transfer; in the other 50% of the sample, farmers are rewarded with a picture‐based insurance policy that has an equal value as the cash transfer.

2 Results from unweighted regressions are qualitatively similar and are available upon request.

3 We find that a change in harvest date is not associated with yields; farmers who adjusted harvest timing produced a statistically insignificant 5.4 quintals less for tomato and 0.3 quintals more for wheat than farmers whose harvest dates were not affected.

4 We modified three questions of the HFIAS for this purpose, focusing on the food security dimensions that we expected to be most relevant for our study population. In particular, "Cannot afford sufficient quantity" relates to a question about not being able to "eat enough food" due to a lack of resources; "Cannot afford sufficient variety" relates to a question about not being able to "eat enough different kinds of food to get a balanced diet" due to a lack of resources; and "Cannot access sufficient variety" relates to a question about not being able to "find the foods that you wanted in the market because these different kinds of food weren't there or prices had gone up".

5 Wheat farmers were significantly more likely to report food insecurity before the lockdown; 1.3% of these farmers could not afford a sufficient quantity of food; 3.4% could not afford a sufficient variety of food; and 6.2% could not access a sufficient variety of food, with negligible numbers of tomato farmers reporting such food insecurity. Such differences are likely related to pre‐existing differences between farmers of these crops. Since tomato farmers reported higher levels of food security already before the lockdown, our analyses focus on changes in food insecurity over time, that is, the variable that indicates that a farmer became food insecure during, but not before, the lockdown.

6 For brevity, we are aggregating these variables across different types of disruptions. Separating income reductions from factors during the production and harvesting phase from income reductions due to issues during the post‐harvest and marketing phase yields very similar results.

7 We estimate a linear probability model for binary variables. Coefficients estimated using probit and logit models are similar in direction and significance.

8 We do not have values for all observations, since these variables are acquired from farmers’ KisanCam data, and not all farmers participated in KisanCam during the rabi season. We therefore impute missing values and include as a control a dummy variable to indicate observations for whom these variables are missing and needed to be imputed.

9 See https://economictimes.indiatimes.com/news/economy/agriculture/direct‐payment‐system‐for‐grain‐farmers‐of‐punjab‐haryana‐soon/articleshow/70686123.cms?from = mdr.

10 Large scale programs exist in the United States, Canada, and the European Union, but these tend to depend on public subsidies and re‐insurance arrangements, have been found to induce distortions in land allocation and resource management, and benefit larger, wealthier farmers (Smith et al., 2018).

11 For instance, there was a one‐time attempt in Haryana in 2018 and Niti Aayog, a prestigious policy think tank in New Delhi, issued an official recommendation to the national government in 2019 to formally include tomato in the MSP scheme, together with other crops such as garlic, onions, and potatoes.

12 The BBY scheme replicates an innovative scheme by the state government of Madhya Pradesh, the Bhavantar Bhugtan Yojana, which introduces the novelty of relying on the market's price—instead of the price at which a given farmers sells—to determine payments. However, the scheme has been shown to be open to high implementation costs associated with the mandatory procurement of produce by official mandis and manipulation by traders to lower market prices and trigger payments (Narayanan and Tomar, 2018; NICR, 2018).
