# Supplementary material for: Crop prices, farm incomes, and food security during the COVID‐19 pandemic in India: Phone‐based producer survey evidence from Haryana State
Source: Agric Econ. 2021 May 25;52(3):525–42. doi: 10.1111/agec.12633 (PMC8207062; doi:10.1111/agec.12633)
Supplement: Supplementary file 1 — Appendix Table 1—Disaggregated food insecurity experiences before and during the lockdown Appendix Table 2—Determinants of attrition in tomato farmers (probit) [file AGEC-52-525-s001.docx]

**Reduced crop income during the COVID-19 pandemic in India: The perils of price risk**

**Online Appendix**

Francisco Ceballos^1^ | Samyuktha Kannan^2^ | Berber Kramer^1^

^1^ International Food Policy Research Institute (IFPRI), Markets, Trade and Institutions Division, 1201 Eye Street NW, Washington, DC, 20005, United States.

^2^ International Food Policy Research Institute, South Asia Regional Office. Dev Prakash Shastri Marg, Pusa, New Delhi, Delhi 110012, India.

**Correspondence**

Francisco Ceballos, International Food Policy Research Institute (IFPRI), Markets, Trade and Institutions Division, 1201 Eye Street NW, Washington, DC, 20005, United States.

Email: f.ceballos@cgiar.org

**Appendix Table 1 – Disaggregated food insecurity experiences before and during the lockdown**

|  | | Wheat producer | | | | Tomato producer | | | | | Difference | | | |
| --- | --- | --- | --- | --- | --- | --- | --- | --- | --- | --- | --- | --- | --- | --- |
|  | | Mean | | Std. dev. | | | Mean | | Std. dev. | | Mean | | Std. err. | |
|  | | (1) | | (2) | | | (3) | | (4) | | (5) | | (6) | |
|  | |  | |  | | |  | |  | |  | |  | |
| Any food insecurity experience | |  | |  | | |  | |  | |  | |  | |
| Before the lockdown | | 0.075 | | 0.263 | | | 0.002 | | 0.045 | | -0.073*** | | 0.012 | |
| During the lockdown | | 0.268 | | 0.443 | | | 0.196 | | 0.397 | | -0.072** | | 0.023 | |
|  | |  | |  | | |  | |  | |  | |  | |
| Could not afford sufficient quantity of food | |  | |  | | |  | |  | |  | |  | |
| Before the lockdown | | 0.013 | | 0.113 | | | 0.000 | | 0.000 | | -0.013* | | 0.005 | |
| During the lockdown | | 0.011 | | 0.105 | | | 0.000 | | 0.000 | | -0.011* | | 0.004 | |
|  | |  | |  | | |  | |  | |  | |  | |
| Could not afford sufficient variety of food | |  | |  | | |  | |  | |  | |  | |
| Before the lockdown | | 0.034 | | 0.183 | | | 0.000 | | 0.000 | | -0.034*** | | 0.007 | |
| During the lockdown | | 0.046 | | 0.209 | | | 0.004 | | 0.064 | | -0.042*** | | 0.010 | |
|  | |  | |  | | |  | |  | |  | |  | |
| Could not **access** sufficient variety of food | |  | |  | | |  | |  | |  | |  | |
| Before the lockdown | | 0.062 | | 0.241 | | | 0.002 | | 0.045 | | -0.054*** | | 0.010 | |
| During the lockdown | | 0.250 | | 0.433 | | | 0.194 | | 0.396 | | -0.060* | | 0.021 | |
|  | |  | |  | | |  | |  | |  | |  | |
| Number of observations | 1,162 | |  | | 483 | | |  | |  | |  | |  |

*Note:* Percentage of farmers that reported experiencing a given food insecurity experience ‘rarely’, ‘often’, or ‘frequently’ during the period of a month. The food security questions were asked for two periods: (i) any point during the month before the lockdown and (ii) any point during the month before the interview (during the lockdown period); respectively referred to as ‘before the lockdown’ and ‘during the lockdown’. Column (5) indicates statistical significance from unpaired *t*-tests for differences between wheat and tomato farmers, * *p* < 0.05, ** *p* < 0.01, *** *p* < 0.001.

**Appendix Table 2 – Determinants of attrition in tomato farmers (probit)**

|  | Farmer did not complete last survey (attrited)  (1) |
| --- | --- |
| Farmer age - lowest tercile (18-35 years) | 0.382* |
|  | (0.154) |
| Farmer age - highest tercile (49-83 years) | 0.212 |
|  | (0.163) |
| Medium education level | -0.042 |
|  | (0.132) |
| High education level | -0.234 |
|  | (0.182) |
| Above median landholdings [0-1] | 0.215 |
|  | (0.162) |
| Transplanting date | 0.003* |
|  | (0.001) |
| Harvested later than median farmer | -0.050 |
|  | (0.147) |
| Reported difficulty accessing inputs in first survey | -0.232 |
|  | (0.195) |
| Reported spending more on labour in first survey | 0.283 |
|  | (0.194) |
| Reported spending more on machinery in first survey | -0.768*** |
|  | (0.228) |
| Reported borrowing to cope with loss in first survey | 0.362 |
|  | (0.266) |
| Reported receiving compensation in first survey | 0.944 |
|  | (0.803) |
| Constant | -58.44* |
|  | (26.02) |
|  |  |
| N | 611 |
| Pseudo R squared | 0.083 |

*Note:* Sample includes only tomato farmers with dependent variable taking a value of 1 if the farmer did not complete the last survey and 0 if he remained in the sample. Other controls include block fixed effects. Standard errors clustered by village. * *p* < 0.05, ** *p* < 0.01, *** *p* < 0.001.
